# Heat Killed *Salmonella typhimurium* Protects Intestine Against Radiation Injury Through Wnt Signaling Pathway

**DOI:** 10.1155/2021/5550956

**Published:** 2021-06-18

**Authors:** Yuanyuan Chen, Kun Cao, Hu Liu, Tingting Liu, Lei Liu, Hongran Qin, Zebin Liao, Xuguang Hu, Bailong Li, Cong Liu, Jianming Cai, Jianguo Cui, Fu Gao, Yanyong Yang

**Affiliations:** ^1^Department of Radiation Medicine, Faculty of Naval Medicine, Naval Medical University, Shanghai, China; ^2^Department of Nuclear Radiation, Shanghai Pulmonary Hospital, Tongji University, Shanghai, China; ^3^Department of Radiation Medicine, Beijing Institute of Radiation Medicine, Beijing, China; ^4^Department of Gastrointestinal Surgery, Changhai Hospital, Shanghai, China

## Abstract

Gastrointestinal (GI) toxicity caused by ionizing radiation (IR) is a dose limiting factor in radiotherapy and a great threat for individual nuclear-related military missions. However, there are currently no available strategies to effectively prevent the damage on the intestine induced by IR. In the present study, the protective activity of Heat Killed *Salmonella typhimurium* (HKST) on intestine against IR was investigated. Through mouse intestinal organoids and whole body irradiation of mice, we found that the pretreatment with HKST significantly preserved the structure of small intestine upon IR exposure and promoted the proliferation of intestinal cells post-IR. Further study revealed that the radioprotective effects of HKST were involved in DNA damage response (DDR) signaling. Moreover, the stimulation of DDR signaling by HKST upon radiation damage was mediated by Wnt signaling, in which the inhibition of Wnt signaling diminished the radioprotective effects of HKST. To sum up, our study suggested HKST as a potential radioprotectant used for prevention of IR-induced GI toxicity.

## 1. Introduction

Patients received radiotherapy and victims from nuclear accidents unavoidably suffer from the damage induced by ionizing radiation (IR) [1]. Acute radiation sickness, which is also known as acute radiation syndrome (ARS), often occurs after a sudden high dose of IR exposure. At a dose less than 6 Gy, the injuries are primarily as a result of hematopoietic syndrome that can be prevented by the bone marrow shielding or cured by transplantation of bone marrow [2, 3]. Gastrointestinal (GI) toxicity (also known as the GI syndrome) is primarily induced by a higher dose of radiation, in which a mass of intestinal stem cells (ISCs) is irreparably suppressed, the regeneration of villi is severely impaired, and the epithelial integrity along the entire GI tract is compromised [4]. Victims with GI syndrome usually bear the pain of fluid loss, malabsorption, and electrolyte imbalances [5]. Besides, the invasion of enteric pathogens and flora into the bloodstream due to the epithelial integrity damage could lead to sepsis and death [6]. Unfortunately, there are currently no medical countermeasures approved to prevent or ameliorate GI syndrome [7]. Although a handful of Food and Drug Administration (FDA) approved radioprotectors were reported to eliminate internally ingested radiation or scavenge free radical species, the unfavorable side effect profiles limit the treatment of patients on a large scale [8, 9]. There is a great need for agents to diminish radiation-induced injury on the intestine.

Toll-like receptors (TLRs) agonists represent a series of effective agents in facilitating the nullification of the IR-induced injuries. Since TLR5 was demonstrated to counteract radiation-induced damage on mice and monkey [10], the activation of other TLR family members, including TLR2/6, TLR4, and TLR9, has been reported to effectively alleviate irradiation-induced damage [11–13]. Nevertheless, as different TLRs distribute among various organs and initiate different signaling pathways, the radioprotective effects by coactivating multiple TLRs provide better protective effects than the stimulation of single TLRs. The evidence comes from the fact that *Escherichia coli* O111: B4 LPS, the coagonist of TLR4 and TLR2, exerts stronger immune stimulation effects than that from TLR2 or TLR4 agonist used alone or in combination [14].

Heat Killed *Salmonella typhimurium* (HKST) represents an potent coagonist of TLR2 and TLR4 receptors, and immune system of mammalian cells can be stimulated, as *Salmonella typhimurium* is recognized by multiple TLRs [15, 16]. In our preliminary work, damage to bone marrow, spleen, and testis, which were induced by *γ*-irradiation, could be significantly made easy by the pretreatment of HKST [17]. However, the radioprotective effects of HKST on small intestine have remained unclear. In the current study, HKST was demonstrated to alleviate the radiation-induced injury on small intestine in vitro and in vivo, which was further confirmed to be in a Wnt signaling dependent manner.

## 2. Materials and Methods

### 2.1. Medium and Reagents

HKST was purchased from InvivoGen Asia (Hong Kong, China) and suspended in phosphate buffered saline (PBS). PBS and RMPI1640 medium were obtained from Hyclone (Logan, UT). ICG-001 was obtained from Selleck Chemicals (Shanghai, China). IntestiCult Organoid Growth Medium (Mouse) was purchased from STEMCELL Technologies Inc. (Canada). Growth factor-reduced Matrigel was purchased from Corning Incorporated (USA) and stored in −20°C.

### 2.2. Mouse Intestinal Organoids Culture and Treatment

Mouse intestinal crypts isolation and intestinal organoids culture were performed as previously described with modest modification [18]. Briefly, small intestine was obtained after mice was euthanized with CO_2_ and then washed with PBS followed by being longitudinally opened. Intestinal crypts were dissociated with PBS plus EDTA (15 mm). 250 crypts/well were suspended in the mixture composed of 75% growth factor-reduced Matrigel and 25% IntestiCult Organoid Growth Medium. Enough medium was supplemented after the Matrigel polymerized at cell incubator (37°C, 5% CO_2_). For HKST treatment, HKST was dissolved in IntestiCult Organoid Growth Medium (10^7^ cells/ml), and the medium was replaced every 3 days. Optical image was taken after 7 days of cultivation.

### 2.3. Cell Treatment

Cells (human intestinal epithelial cells (HIEC), ATCC) were maintained in 1640 medium with 1% antibiotics from Gibco and 10% fetal bovine serum (Gibco, Australia) at 37°C in a 5% CO_2_ cell incubator. Cells were pretreated with HKST (10^7^ cells/ml) alone or in combination with ICG-001 (25 *μ*M) at 12 hours prior to IR.

### 2.4. Mice and Treatment

Animal experiments were approved by the Ethic Committee of The Second Military Medical University according to the instruction about Care and Use of Laboratory Animals published by the US NIH (Publication No. 96-01). Mice (C57BL/6 J, Male, 6–8 weeks, Jihui Experimental Animal Breeding Co., Ltd) were housed in a room with a 12 h light/dark cycle, in which water and food were obtained ad libitum. HKST (InvivoGen, US, Lot: HST-39-01) resuspended in PBS (10^7^/mice) was administrated through gavage 12 hours prior to irradiation.

### 2.5. Irradiation

Mice (in transparent boxes) and HIEC cells were exposed to *γ*‐radiation (^60^Co source, Second Military Medical University, China). The dosage was set as follows: whole body radiation of 8 Gy, a dose rate of 1 Gy/min. Mouse intestinal organoids were irradiated with 0 Gy, 4 Gy, and 6 Gy.

### 2.6. Histopathology and Immunohistochemistry

Mice were randomly divided into control group, ionizing radiation (IR) group, IR plus HKST (IR + HKST) group, IR plus ICG-001 (IR + ICG-001) group, and IR plus HKST in combined with ICG-001 (IR + HKST + ICG-001) group; each group contained at least 3 mice. Mice were anesthetized with pentobarbital sodium (60 mg/kg) through intraperitoneal injection and pretreated with HKST (10^7^ cells/mice), ICG-001 (10 mg/kg), HKST (10^7^ cells/mice) in combination with ICG-001 (10 mg/kg), or placebo at 12 hours before 8 Gy IR exposure. At 3 days postirradiation, mice were sacrificed; small intestine was then isolated and fixed in paraformaldehyde and subjected to histological and immunohistochemical examination. Hematoxylin and eosin (H&E) staining was employed for the pathological morphology evaluation, in which the tissue slices were 3 *μ*m. To detect the apoptosis rate in the small intestine, TdT-mediated dUTP Nick-End Labeling (tunel) assay was employed according to the manufacture instructions of the tunel kit (Roche, Basel, Switzerland). For the immunohistochemistry examination, tissue slices were stained with Olfm4 antibody (Cell Signaling Technology, Inc), and then the biotinylated secondary antibodies and DAB substrate kit were employed for immunohistochemistry.

### 2.7. Immunofluorescence Assay

Cell-Light EdU DNA cell proliferation kit (C103102, RiboBio) was employed for the EDU staining. Briefly, intestinal organoids were cultured with EdU (25 mm) for 1.5 h at 37°C, after which the organoids were fixed with 4% paraformaldehyde and permeabilized with 0.5% Triton X-100. And then, the organoids reacted with 1 × Apollo cocktail (RiboBio). For immunofluorescence analysis, tissue slices were stained with antibody against ki67 (Abcam, USA), *ß*-catenin (Abcam), and phosphorylated *P*65 (Proteintech Group, China), and then the secondary antibodies were employed. The images of tissues were obtained using a fluorescent microscope (Olympus BX60, Center Valley, PA, USA) equipped with a digital camera (Retiga 2000 R, Surrey, BC, Canada).

### 2.8. Western Blot Analysis

M‐PER Mammalian Protein Extraction Reagent was employed to extract proteins from irradiated cells at 8 hours post-IR exposure. Proteins were separated by SDS-polyacrylamide gel electrophoresis and transferred to PVDF membranes. The antibodies used to probe the membranes were listed as follows: GAPDH (1 : 1000) was purchased from Proteintech (China); phosphorylated‐ATR (1 : 1000), ATR (1 : 1000), ATM (1 : 1000), and phosphorylated‐ATM (1 : 1000) were purchased from Cell Signaling Technology (USA); CHK1 (1 : 1000), phosphorylated‐CHK1 (1 : 1000), phosphorylated‐CHK2 (1 : 1000), CHK2 (Abcam, 1 : 1000), MyD88 (CST, 1 : 1000), and *p*21 (1 : 1000) were purchased from Abcam (USA). Levels of proteins were done by an ECL western blotting detection system (Thermo Fisher Scientific, Waltham) after the specific secondary antibodies (Servicebio, China) were probed.

### 2.9. Statistical Analysis

Data of each experiment are presented as means ± the standard error of mean (SEM). Student's *t* test was employed to evaluate the significant differences, in which *P* values less than 0.05 were considered significant. All the experiments were repeated for at least 3 independent times, and the quantification was conducted in a blinded fashion.

## 3. Results

### 3.1. HKST Protected Radiation-Induced Intestinal Injury in Mice

**Figure 1:**
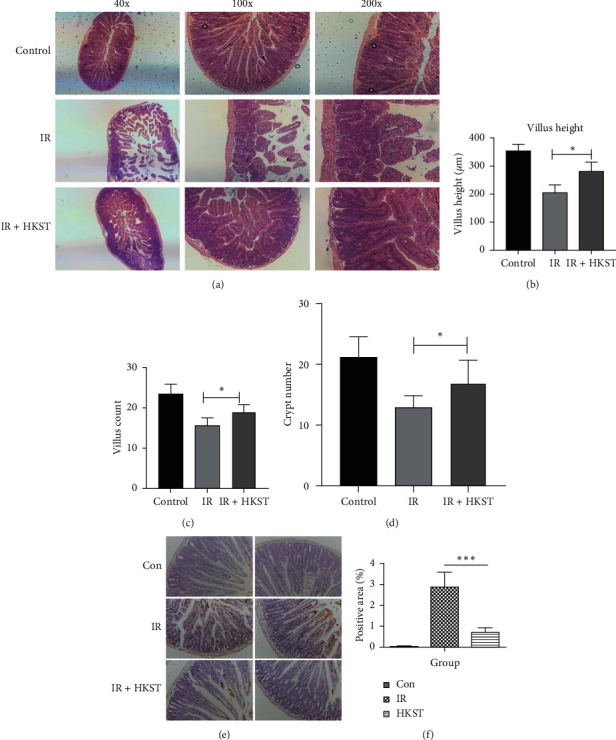
Mice intestine damage induced by radiation exposure was alleviated by HKST pretreatment. Mice receiving IR exposure were pretreated with PBS or HKST. At 3 days postirradiation, mice were sacrificed, and the protective effect of HKST on intestine was tested by Hematoxylin-Eosin (HE) staining (a), based on which villus length (b), crypt depth (c), and number of crypts (d) were analyzed. TdT-mediated dUTP nick-end labeling (TUNEL) assay was performed in intestine tissues, and quantification analysis was done with image J software (e, f). Values were given as mean ± SEM (*n* = 6), ^*∗*^*P* < 0.05, ^*∗∗∗*^*P* < 0.001 represents the HKST + IR group versus IR group.

When exposed to a high dose of radiation, gastrointestinal toxicity remains a critical clinical issue to be settled. Thus, the radioprotective effect of HKST on intestine was investigated using whole body irradiation of mice. As shown in Figure 1(a), after radiation exposure, the structure of intestine was severely damaged, in which the villus became shorter, and the villus cells of the small intestine became discontinuous. HKST pretreatment effectively preserved the structure of intestine upon IR exposure, in which the villus length, crypt depth, and the number of crypts were significantly improved (Figures 1(a)–1(d)). Accordingly, increased apoptosis rate in the small intestine was induced upon IR exposure, and HKST pretreatment significantly decreased the apoptosis rate as compared to the IR group (Figures 1(e) and 1(f)).

### 3.2. HKST Promoted Proliferation of Intestinal Cells In Vitro and In Vivo Postirradiation

**Figure 2:**
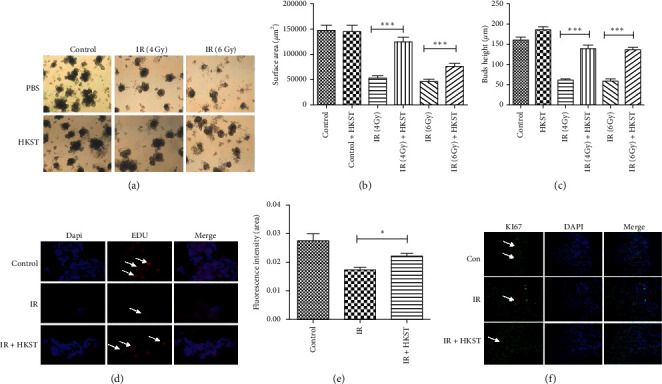
HKST promoted proliferation in intestinal organoids and in vivo postirradiation. Intestinal crypts were isolated from mice small intestine and then seeded into Matrigel. Upon IR exposure, photos of intestinal organoids were taken after intestinal crypts were cultured after 7 days (a). Surface area and bud height of intestinal organoids were measured with image J software and analyzed with GraphPad prism 5, in which 30 organoids were calculated for each group (b, c). Values were given as mean ± SEM, ^*∗∗∗*^*P* < 0.001 represents the HKST + IR group versus IR group. The proliferation of intestinal organoids and crypts was detected by immunofluorescence staining of EDU (d) and Ki67 (f), respectively. The fluorescence intensity of EDU in organoids was measured by image J software and analyzed with GraphPad prism 5 (e). Values were given as mean ± SEM (*n* = 6), ^*∗*^*P* < 0.05 represents the HKST + IR group versus IR group.

Intestinal organoids were primarily established to mimic the regeneration of intestinal crypts in vitro [18, 19]; we, therefore, detected whether the pretreatment of HKST could protect the intestinal organoids against IR-induced injury. As our result showed that the structure of intestinal organoids was destroyed by IR, in which the surface area of organoids and the height of organoids' buds were dramatically decreased upon IR exposure as compared to the control group, the destructive effect on organoids was elevated as the dosage of IR increased (Figures 2(a)–2(c)), whereas the structure of intestinal organoids was effectively preserved by HKST pretreatment upon IR damage, in which the budding number and surface area of organoids in the HKST-treated group were more striking than those of the IR group (Figures 2(a)–2(c)), indicating the protective effects of HKST on intestinal organoids against IR injury. With immunofluorescence staining, we found that the proliferation of cells in organoids (Edu positive) and crypts of mice (Ki67 positive) were inhibited in IR group, and the HKST pretreatment significantly promoted the proliferation of intestinal cells as compared to IR group (Figures 2(d)–2(f)).

### 3.3. Pretreatment with HKST Stimulates DNA Damage Response Upon IR Exposure

**Figure 3:**
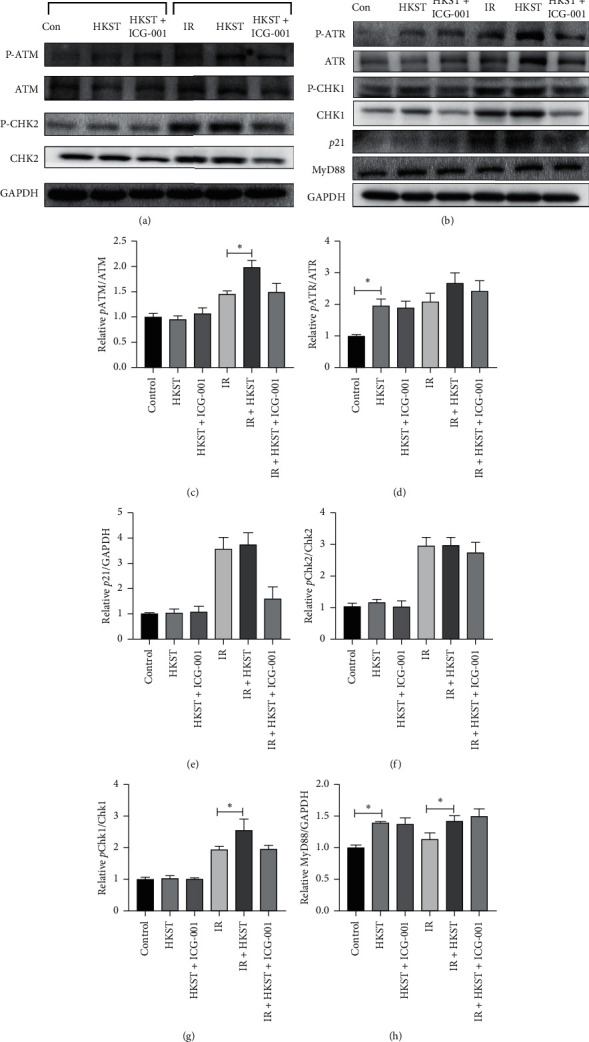
HKST treatment promoted the activation of DDR pathway, and the stimulated activity was diminished by ICG-001. HIEC cells were pretreated with HKST alone or in combination with ICG-001 for 12 h prior to radiation exposure (8 Gy). Proteins were extracted at 8 hours postradiation, and then the levels of phosphorylated‐ATR (*P*-ATR), ATR, phosphorylated‐CHK1 (*P*-CHK1), CHK1, phosphorylated‐ATM (*P*-ATM), ATM, phosphorylated‐CHK2 (*P*-CHK2), CHK2, *p*21, and MyD88 were detected with Western blot (a, b). The levels of *P*-ATM (c), *P*-ATR(d), *p*21(e), *P*-CHK2 (f), *P*-CHK1(g), and MyD88 (h) were measured by image J software and analyzed with GraphPad prism 5. ^*∗*^*P* < 0.05.

DNA damage response (DDR) is an essential mode for mammalian cells to maintain and modulate the genome integrity, thereby nullifying IR-induced toxicity [20, 21]. HIEC cells were employed to detect the effect of HKST on DDR upon radiation exposure. Our study showed that levels of *P*-ATM, *P*-ATR, and *P*-Chk1 were significantly increased in HKST-treated cells (Figures 3(a)–3(d) and 3(g)). However, HKST showed minor effects on the phosphorylation of Chk2 and *p*21 expression level (Figures 3(e) and 3(f)). With selective inhibitor of Wnt signaling named ICG-001 [22], the mechanism involved in the stimulation of DDR by HKST after IR exposure was determined. MyD88 is a direct mediator in the downstream of TLR, and then we checked the expression of MyD88 in HSKT treated cells. It was observed that HKST significantly increased the level of MyD88 in unirradiated cells and irradiated cells, which was not influenced by ICG-001 treatment (Figures 3(b) and 3(h)). These data suggested that TLR mediated activation of MyD88 is not dependent on Wnt signaling pathway.

### 3.4. Radioprotective Effects of HKST on Intestine Was Blocked by Wnt Signaling Inhibitor

**Figure 4:**
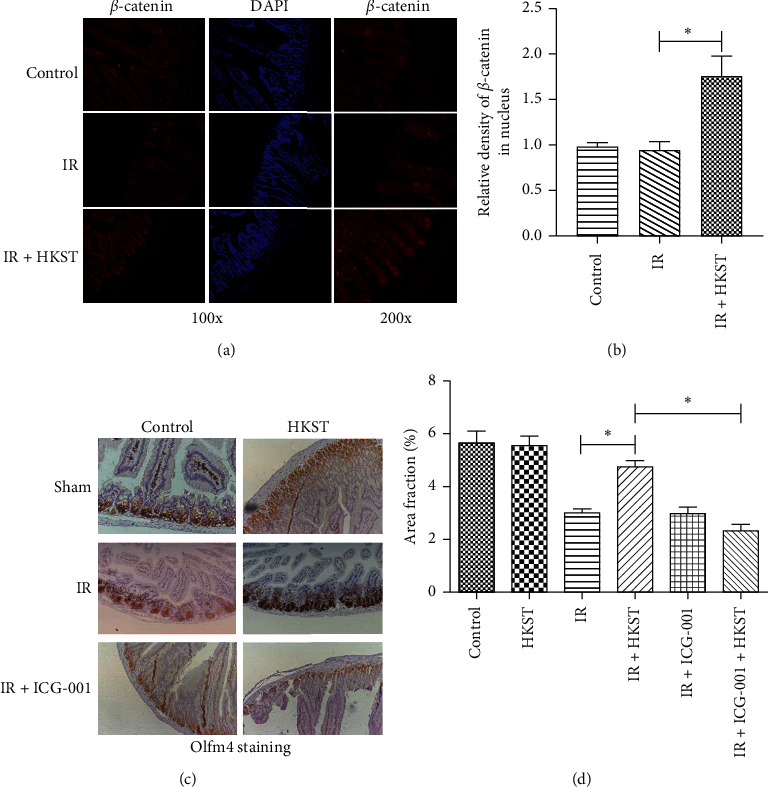
IR-induced damage on intestinal stem cells was alleviated by HKST, and the preserved effect was diminished by ICG-001. Small intestines isolated from mice untreated or receiving IR exposure plus agents were sectioned into 3 *μ*m, and immunohistochemistry was employed to detect the levels of *ß*-catenin (a) and Olfm4 (c). The nuclear density of *ß*-catenin was quantified with image J software and analyzed (b). The positive area of Olfm4 staining cells was measured by Image J software and analyzed (d). Values were given as mean ± SEM (*n* = 6), ^*∗*^*P* < 0.05.

The intestinal stem cells that are highly susceptive to IR damage represent the sources of intestine regeneration. To determine whether HKST affects Wnt signaling, we checked the cellular localization of *ß*-catenin in mice intestine isolated from different groups. We found that the nuclear localization of *ß*-catenin was upregulated in HKST treated mice (Figures 4(a) and 4(b)), suggesting the activation of Wnt and downstream factors. Olfm4 is a potent marker of murine intestinal stem cells in mice intestine [23]. In our result, the level of Olfm4 in intestine was notably decreased upon IR injury as compared to the control group (Figures 4(c) and 4(d)), whereas the HKST pretreatment preserved the levels of intestinal stem cells, which again confirmed the protective effect of HKST on intestine against radiation damage. However, after ICG-001 treatment, the radioprotective activity of HKST on intestinal stem cells was significantly blocked (Figures 4(c) and 4(d)), indicating that the radioprotective of HKST on intestine was blocked by Wnt signaling inhibitor.

### 3.5. HKST Activated Target Cells in Liver and Spleen besides Intestine

**Figure 5:**
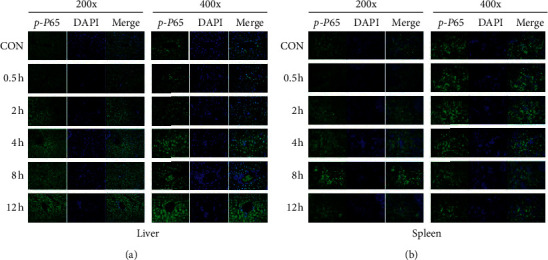
NF-*κ*B signaling in liver and spleen was activated by HKST treatment. Mice untreated or treated with HKST were sacrificed, and the liver and spleen were collected. The activation of NF-*κ*B nuclear translocation in liver (a) and spleen (b) was detected by immunofluorescence staining of phosphorylated *P*65 (*P*-*P*65).

Distinct TLR distributes in different tissues, which suggests that the radioprotective effects of HKST could be an integrated effect. Besides intestine, we investigated whether HKST activates other cells, as most TLRs activate NF-*κ*B as a downstream signaling pathway. To identify target cells of HKST, we perform a *p*65 staining in liver, spleen, and bone marrow, which are tissues with high level of TLRs. Our data showed that, upon HKST treatment, *p*65 expression and translocation were strongly activated in liver and spleen (Figures 5(a) and 5(b)). These findings suggest that the protective effects of HKST on intestine are not just a local activation of DNA damage repair in intestine, but also a whole body protective effects.

## 4. Discussion

Intestinal epithelium is fast self-renewing tissue in mammals and is, therefore, sensitive to IR damage. IR-induced GI toxicity is a limited factor in abdominal pelvic radiotherapy and a great threat to public health in the potential nuclear accident [24]. Nonetheless, there are no effective strategies approved to alleviate the pain of victims with GI syndrome, and the currently reported agents have unfavorable side effects [8, 9]. The development of novel agents protecting against IR-induced intestinal damage is of great significance. Currently, the radioprotective effects of HKST on small intestine were investigated. As shown in our results, the radiation-induced damage on intestine was dramatically mitigated by pretreating with HSKT in vitro and in vivo, in which the intestinal organoids and the structure of small intestine were effectively protected with HKST treatment, and HKST pretreatment remarkably promoted the proliferation of intestinal cells post-IR exposure (Figures 1 and 2). HKST is a potent coagonist of TLRs family, which has been reported to alleviate the radiation-induced injury on bone marrow, spleen, and testis in our previous study [17]. It has been demonstrated that the attenuated *Salmonella typhimurium* could be employed to stimulate immune system or developed into vaccine thereby protecting against malaria or tumor [25, 26]. Besides, the therapeutic use of bacteria in preventing IR-induced GI toxicity has begun to receive more attention [27–29]. Our study demonstrates that HKST is of great potential alleviating injury on intestine induced by radiation and facilitating the relief of victims with GI syndrome in clinical practice.

Generally, the main critical target of IR is considered to be DNA [30]. Upon IR exposure, the irreparable DNA damage is induced, and the unrepaired or error-repaired DNA damage subsequently leads to cellular damage or death [31]. DNA double-strand breaks (DSBs) are regarded as the most deleterious form of DNA damage, which can result in chromosomal aberrations, loss of genetic materials, cell death, or other detrimental consequences [32]. In response to DSB, homologous recombination (HR) and nonhomologous end joining (NHEJ) are two main modes to repair DSBs in mammalian cells, in which ATR and ATM are confirmed to be the key components [20, 21]. As DDR is critical for the maintenance and modulation of genome integrity in intestinal cells after radiation exposure [33, 34], we speculated that the radioprotective effects of HKST might be involved in DDR. Our results show that, after radiation exposure, levels of *P*-ATM, *P*-ATR, and *P*-CHK1 were significantly increased in cells pretreated with HKST (Figure 3). The dysfunction of genome integrity caused by radiation damage requires DDR to prevent the transmission of incompletely replicated or damaged chromosomes. DNA damage repair reactions in mammalian cells require the cell cycle arrest by the activation of a DNA damage checkpoint [35]. ATM and ATR are the main regulators of two major checkpoint pathways, in which ATR‐CHK1 checkpoint signaling is essential for HR repair pathway [36, 37]. In some conditions, the cell-cycle arrest was initiated by ATR-CHK1 signaling pathway [38]. There was a minor change on *P*-CHK2, between HKST treatment and IR group, and the ATR pathway might be more crucial in facilitating the protective effects of HKST against IR.

The maintenance and self-renewal of intestinal cells, especially the stem cells, in adult mammalian require Wnt signaling pathway, whereas the inactivation of the essential components of Wnt signaling leads to the defect in intestine development and regeneration [39, 40]. Previous studies have demonstrated that DDR activation was amplified by Wnt signaling in mammalian cells, whereas the inhibition of Wnt signaling fails to resolve DSBs after radiation [41–43]. As the protective activity of HKST against radiation injury was confirmed to be related to DDR, and the pretreatment of HKST effectively preserved intestinal stem cells when exposed to IR (Figure 4), the radioprotective effect of HKST might be mediated by Wnt signaling pathway. As expected, the stimulation of ATR and ATM signaling upon IR in HKST-treated cells and the radioprotective effects of HKST on intestinal stem cells in mice were diminished by the selective inhibitor of Wnt signaling pathway (Figures 3 and 4). Previous studies have shown that the kinases ATM/ATR were involved in the inflammatory responses induced by TLRs signaling [44], and TLRs signaling was reported to modulate the DNA repair [45]. We also checked the expression of MyD88, a key mediator in the downstream of TLR signaling pathway. Our data showed that HKST increased the level of MyD88, which was unaffected by ICG-001, showing that TLR-MyD88 activation is in the upstream of Wnt and DDR activation. As HKST is a coagonist of TLRs, the role of TLRs signaling in DDR mediated by Wnt signaling pathway upon IR damage remains to be investigated further. As distinct TLRs family are expressed in different organs, we presumed that HKST might activate other cells, and the protective effects of HKST against IR result from whole body protective effects.

## 5. Conclusion

Gastrointestinal (GI) toxicity caused by ionizing radiation (IR) appears to be a limited factor in radiotherapy and a great threat for those exposed to high dose radiation. However, there are currently no available strategies to effectively prevent or mitigate the damage on intestine induced by IR. Our study demonstrated that Heat Killed *Salmonella typhimurium* (HKST) could significantly alleviate the damage on intestine induced by IR, and the radioprotective effect of HKST depended on DDR mediated by Wnt signaling pathway. Together, our study provides a potential strategy for IR-induced GI toxicity prevention. [46]

## Data Availability

The data used to support the findings of this study are included within the article.
